# Physicochemical Evolution of Glutinous Rice Flour and Its Influence on Tangyuan Processing Performance

**DOI:** 10.3390/foods15101789

**Published:** 2026-05-18

**Authors:** Fengzhang Wang, Ning Li, Jianing Dou, Enhong Gao, Litao Tong

**Affiliations:** 1 Institute of Food Science and Technology, Chinese Academy of Agricultural Sciences, Beijing 100193, China; wangfengzhang@caas.cn (F.W.); maillining@163.com (N.L.); dou11140029@163.com (J.D.); 2 Chengdu Agricultural Science and Technology Center, Chinese Academy of Agricultural Sciences, Chengdu 610000, China; gaozyzx@163.com; 3 Zhongyuan Research Center, Chinese Academy of Agricultural Sciences, Xinxiang 453500, China

**Keywords:** glutinous rice flour, Tangyuan, texture, microstructure, processing

## Abstract

The quality of glutinous rice flour (GRF) plays a critical role in determining the processing suitability of Tangyuan, a traditional Chinese glutinous rice-based food. This study systematically characterized the physicochemical, pasting, textural and digestive properties of twelve representative GRFs, as well as the cooking behaviors of the resulting product of Tangyuan. The results revealed that different GRFs displayed varied protein, total starch and amylopectin contents. The microstructure of Tangyuan exhibited three main types: robust and dense (e.g., H1 and H3), porous and soft (e.g., S1 and S4), and fragmented and disrupted (e.g., S2 and S3) networks. Rheological and pasting profiling revealed that doughs with extreme rigidity (G′ > 10^4^ Pa) and high setback values exhibited rapid retrogradation, leading to severe frost cracking during cold-chain storage. Tangyuan with moderate G′, loss tangent (tan δ) below 0.35, and balanced peak viscosities provided optimal viscoelasticity for both mechanical machinability and freeze–thaw stability. Furthermore, S5 with naturally high resistant starch contents significantly attenuated the hydrolysis index, successfully shifting Tangyuan from a high-glycemic to a medium-glycemic profile. The results provide valuable insights into the screening of raw glutinous rice flour from different origins, offering theoretical guidance for the standardized production of freeze–thaw-stable and low-glycemic functional Tangyuan.

## 1. Introduction

Glutinous rice flour (GRF) is a complex, heterogeneous matrix primarily composed of key characteristic components such as starch, proteins, and endogenous lipids. As the core raw material, it is indispensable for the formulation of traditional rice-based products and modern functional foods [1]. The nutritional efficacy, flavor profile, and overall eating quality of glutinous rice products are fundamentally governed by the relative content, and multi-scale microstructure of these components, as well as their physicochemical evolution during food processing [2]. Tangyuan (traditional sweet dumplings), a highly representative glutinous rice product, is predominantly manufactured using commercial GRF or modified glutinous materials. The modern industrialization of Tangyuan relies on a standardized operational workflow, encompassing flour hydration (dough formation), filling encapsulation, mold shaping, and ultra-low-temperature rapid freezing [3].

In China, driven by the booming frozen food industry and advancements in cold-chain logistics, Tangyuan has rapidly transitioned from a traditional festive delicacy into a mass-consumed daily product due to their unique soft and sticky texture, representing a massive and continuously growing market share [4]. Currently, most manufacturers employ uniform processing parameters but lack standardized and scientific evaluation systems for raw GRF materials. Consequently, industrial Tangyuan frequently suffers from severe quality defects during freezing and prolonged cold-chain storage, such as structural collapse and surface cracking (frost cleft), skin breakage, excessive cooking loss (leaching/turbid soup), and rapid retrogradation (textural hardening) during terminal boiling [5,6]. These prevalent industrial defects severely restrict the standardized and high-quality development of the Tangyuan industry.

The quality fluctuations of Tangyuan lie in the strategic selection and suitability of the raw materials. The processing suitability of GRF is highly variable and significantly influenced by its botanical and geographical background. Glutinous rice cultivated across different geographical origins and varying cultivars exhibits profound disparities in inherent physicochemical attributes—such as amylopectin chain length distribution, damaged starch content, protein network cross-linking, and pasting properties [7,8]. These intrinsic structural differences critically affect the hydration kinetics and rheological properties of the dough, directly dictating whether the flour can adapt to the intense mechanical shear and thermodynamic stresses of industrial processing.

Currently, while the heterogeneity in Tangyuan quality derived from different flours is well-recognized, systematic frameworks for the processing suitability evaluation of GRF remain insufficient. There is a critical lack of comprehensive studies elucidating how specific flour characteristics—driven by diverse origins and cultivars—specifically affect the processing adaptability of rice products [9,10]. Therefore, a systematic evaluation of GRFs is essential to establish quality criteria specifically tailored for Tangyuan production. In this study, twelve representative GRFs were selected to characterize the physicochemical, pasting, and textural properties and cooking behaviors of the resulting Tangyuan. Such insights will not only unravel the structural–functional mechanisms behind quality variations but also provide robust theoretical guidance for raw material screening, targeted blending, and the standardized industrial production of high-quality dedicated Tangyuan products.

## 2. Materials and Methods

### 2.1. Materials

This experiment used twelve varieties of glutinous rice as the experimental materials. The varieties were selected from Sichuan, Anhui, Henan, and Liaoning provinces in China. We used one variety of Indica glutinous rice (Zhenzhunuo (H3, provided by Xinyang Academy of Agricultural Sciences)) and eleven varieties of Japonica glutinous rice: Xinyounuo 5108 (H1, provided by Xinyang Academy of Agricultural Sciences); Xinyounuo 2308 (H2, provided by Xinyang Academy of Agricultural Sciences); Xinyounuo 9108 (JR-H4, provided by Xinyang Academy of Agricultural Sciences); Weifei Chengdu Glutinous Rice (S1, provided by Chengdu Micangshan E-Commerce Co., Ltd.; Chengdu, China); Sichuan Zigong Round-Grain Glutinous Rice (S2, provided by Baiyibaijie Food Store, Pinduoduo; Shanghai, China); Sichuan Glutinous Rice (S3, provided by Chen’erwa Bean Store, Pinduoduo; Shanghai, China); Sichuan Deyang Glutinous Rice (S4, provided by Chenshiji Firm, Pinduoduo; Shanghai, China); Chengdu Zuichuanwei Glutinous Rice (S5, provided by Chengdu Zuichuanwei E-Commerce Co., Ltd.; Chengdu, China); Chongqing Farmhouse Glutinous Rice (S6, provided by Sichuan Yijia Store, Pinduoduo, Luzhou, China); Northeast Glutinous Rice (D1, provided by Shenyang Xinchang Grain and Oil Trade Co., Ltd., Shenyang, China); and Anhui Xuezao Glutinous Rice (A1, provided by Bengbu Brothers Grain, Oil & Food Technology Co., Ltd., Bengbu, China).

### 2.2. Preparation of Tangyuan

Accurately weigh 25.0 g of glutinous rice flour and place it in a clean container. Add deionized water equivalent to 90% of the dry weight of the glutinous rice flour (i.e., 22.5 g), mix thoroughly, and knead until the dough is uniform and smooth. Then, seal the dough with plastic wrap and rest at room temperature for 20 min. After resting, divide the dough into portions of 3.0 g each, and roll them into balls by hand to prepare raw Tangyuan (glutinous rice dumplings) for subsequent use. Gently place the rolled raw Tangyuan into boiling water, and cook for 4 min under a gently simmering condition.

### 2.3. Chemical Analysis

The samples were analyzed according to GB 5009.5-2025 [11], GB 5009.6-2025 [12] and GB 5009.4-2016 (China) [13] for protein, fat and ash content. The total starch content was determined using a Total Starch Assay Kit (JKY/K-TSTA 07/11, Megazyme International Ltd., Wicklow, Ireland) by the American Association for Clinical Chemistry (AACC)-approved method 76.13. The amylose content was determined using an Amylose/Amylopectin Assay Kit (JKY/K-AMYL 07/11, Megazyme International Ltd., Wicklow, Ireland).

### 2.4. Color Determination

The Hunter L* (lightness), a* (redness) and b* (yellowness) values of Tangyuan were measured using a color difference meter (Chroma Meter CR-400, Konica Minolta, Sensing, Inc., Sakai, Japan) according to [14]. The average value was determined by taking observations from three different locations on a given sample.

### 2.5. Fourier Transformation Infrared (FT-IR) Spectroscopy

A Fourier transform infrared spectrophotometer (Nicolet Is10, Thermo Fisher, Waltham, MA, USA) was used to determine the infrared spectrum of 12 kinds of glutinous rice flour as described by a previous method [15]. The dried glutinous rice flour and potassium bromide (1%, *w*/*w*) were ground into transparent and uniform sheets at the scanned wavelength range of 400–4000 cm^−1^.

### 2.6. Textural Properties

The textural properties were determined as previously described with small modifications [16]. Texture profile analysis (TPA) mode was used with a TAXT 2i/5 texture analyzer (Stable Micro System Ltd., Godalming, UK) and a P/36R probe. Measurements were performed after equilibration at 4 °C for 20 min. The parameters were as follows: descent rate of 2.0 mm/s, test rate of 1.0 mm/s, rise rate of 1.0 mm/s, trigger force of 5.0 g, and compression strain of 50%.

### 2.7. Pasting Properties

GRF samples (moisture content adjusted to 14%, *w*/*w*) were accurately weighed into aluminum RVA canisters, and distilled water was added at a specified ratio to achieve a total solid mass of 3.0 g. The mixtures were thoroughly homogenized using a paddle. The RVA analysis was conducted using the standard starch testing program, which included the following temperature profile: holding at 50 °C for 1 min, heating from 50 to 95 °C at a rate of 12 °C/min, holding at 95 °C for 2.5 min, and cooling from 95 to 50 °C at a rate of 12 °C/min, with a final hold at 50 °C for 2 min. The stirring speed was maintained at 160 rpm throughout the test. Characteristic viscosity parameters, including peak viscosity, trough viscosity, final viscosity, breakdown, and setback, were recorded using the instrument software. Each sample was analyzed in triplicate, and the mean values were reported.

### 2.8. SEM

The microstructure of raw and cooked Tangyuan was determined according to the method of Zhuang et al. [17] with some modifications. All the samples were observed using scanning electron microscopy (SEM, Hitachi S-570, Hitachi, Co., Ltd., Tokyo, Japan) under an acceleration voltage of 10 kV, and at magnifications of 1000× after sputter-coating with gold. Tangyuan was cooked in boiling water for 4 min. Then, the cross-sectional appearance was measured.

### 2.9. Rheological Properties

The rheological properties were determined according to Wang et al. [18] with minor modifications. The samples were measured in a 40 mm diameter plate with 1 mm gap. After conducting a 0.01–10% strain scanning test with a Rheometer (Physica MCR 301, Anton Paar GmbH, Graz, Austria), the oscillation frequency experiment (0.1–10 Hz) was carried out in the linear viscoelastic region with 0.1% target strain. The elastic modulus (G′), viscous modulus (G″) and loss tangent (tan δ) were analyzed.

### 2.10. In Vitro Digestibility

In vitro digestibility was determined by the method from Li et al. [19] with slight modifications. Briefly, the sample was dissolved in distilled water and then heated in a boiling water bath for 30 min. After cooling to 37 °C, 7 glass beads and 20 mL of sodium acetate buffer (pH 5.2) were added into a 50 mL centrifuge tube, and incubated at 37 °C for 20 min. Transfer 300 μL of the mixture to a 5 mL tube, add 3 mL anhydrous ethanol, vortex, and centrifuge at 10,000× *g* for 5 min. Collect supernatant as the 0 min sample. Add 2 mL mixed enzyme solution. At 20, 120, and 180 min, take 0.3 mL digest, add 3 mL anhydrous ethanol, mix, and centrifuge at 10,000× *g* for 5 min. Take 0.1 mL of each supernatant, add 3 mL GOPOD reagent, incubate at 50 °C for 20 min. Transfer 200 μL to a microplate and measure absorbance at 520 nm. Mixed enzyme solution: dissolve 2 × 4.5 g pancreatin in 2 × 40 mL deionized water, vortex, incubate at 37 °C for 10 min, and then centrifuge at 5000× *g* for 10 min.

### 2.11. Statistical Analysis

Significant differences between samples were determined by one-way analysis of variance (ANOVA) with Duncan’s test (*p* < 0.05) using the SPSS software 26.0 (SPSS Inc., Chicago, IL, USA). The data were expressed as means ± standard deviation (SD). Origin 2021 64Bit software was used for charting and principal component analysis (PCA).

## 3. Results and Discussion

### 3.1. Appearance, Surface Morphology, and Cross-Sectional Structure

**Figure 1:**
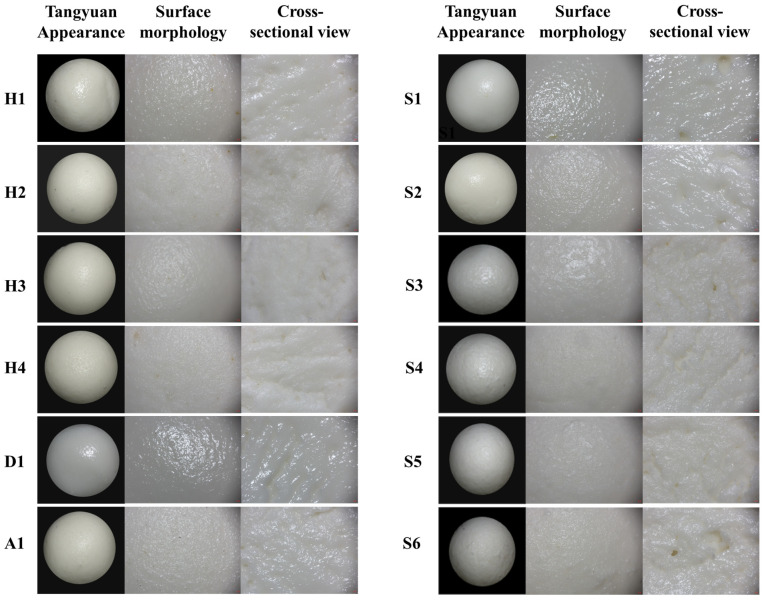
Appearance, surface morphology, and cross-sectional structure of Tangyuan samples. (The images were all taken at a magnification of 2×).

The external characteristics of the Tangyuan samples were evaluated through visual observation of their appearance, surface morphology, and cross-sectional views, as shown in Figure 1. Distinct differences in overall appearance and surface smoothness were observed. H4 and S4 displayed relatively smooth and uniform surfaces, with minimal cracking or deformation. In contrast, H1, S1 and S2 showed increased surface roughness and visible irregularities. The cross-sectional views revealed that H3 and H4 maintained a compact and homogeneous internal structure, while the others exhibited more porous or uneven internal matrices, potentially affecting textural properties and cooking quality. Moreover, D1 presented a notably different surface morphology compared to the H-series samples, characterized by a slightly rougher texture and visible granular features. Its cross-sectional view showed a less uniform internal structure, suggesting differences in hydration or gelatinization behavior during processing. Moreover, S6 showed the highest degree of surface heterogeneity, with obvious aggregated structures and surface fissures. The observed variations in appearance, surface morphology, and cross-sectional structure among the samples reflect differences in ingredient composition, and are likely to correlate with cooking behavior, textural properties, and in vitro digestion kinetics [20].

### 3.2. Chemical Analysis

The proximate composition and starch characteristics of the glutinous rice flour are shown in Table 1. A1, H2, and D1 exhibited the highest total starch contents with no significant differences (*p* > 0.05), whereas H1 showed a significantly lower total starch content at 81.37%. Amylose content, a critical factor determining the physicochemical and processing properties of GRF, remained at extremely low levels (0.29–2.88%) across all groups, which is characteristic of the waxy nature of glutinous rice. H2 recorded the highest amylose content (2.88%), which was significantly higher than the other samples (*p* < 0.05). Conversely, the S-series samples generally displayed lower amylose contents, particularly S1 and S6 at 0.29% and 0.35%, respectively. Accordingly, amylopectin content fluctuated between 76.85% and 86.38%, with D1 presenting the highest concentration and S6 the lowest. These variations in amylose/amylopectin ratios are expected to significantly influence gelatinization behavior, retrogradation, texture, and in vitro digestibility [21]. Regarding lipid profiles, the overall fat content across all groups was relatively low (0.94–1.73%). These differences likely reflect variations in the intrinsic composition of the raw grains, as well as distinct milling effects, such as the varying degrees of bran and germ retention during processing. Additionally, protein constituted the second most abundant component in the flour samples, varying from 6.08% to 6.96%. S4, S5, and A1 contained relatively higher protein levels (6.96%, 6.92%, and 6.92%, respectively) with no significant differences, while H1 and H2 exhibited lower protein contents. It is emphasized that the protein, fat, and amylose contents in glutinous rice flour significantly affect the height-to-diameter ratio, water loss rate, cooking quality, and sensory evaluation of quick-frozen Tangyuan [22].

Moreover, the ash content, reflecting the residual inorganic minerals in the flour, was below 0.35% for all samples, ranging from 0.14% to 0.30%. H3, H4, and D1 showed relatively higher ash contents, whereas S4 exhibited the lowest (0.14%). These differences may indicate variations in the purity of the flour or the addition of mineral-containing ingredients. The observed compositional differences, particularly in amylose/amylopectin ratio and protein content, are likely to influence the technological properties of Tangyuan. Samples with higher amylose content may exhibit different gelatinization and retrogradation behaviors compared to waxy samples (amylose-free), potentially affecting cooking loss, texture, and freeze–thaw stability [23]. Similarly, variations in protein and lipid contents may impact dough handling properties, surface smoothness, and sensory attributes.

### 3.3. Color Analysis

The color parameters (L*, a*, b*) and whiteness values of both raw and cooked Tangyuan samples are demonstrated in Table 2. Significant differences (*p* < 0.05) were observed, reflecting variations in raw material composition and the effects of cooking on color attributes. For raw Tangyuan, the L* values (brightness) ranged from 91.34 (H2) to 96.96 (S5), indicating that all samples exhibited high lightness. The a* values (redness–greenness) for raw samples were negative across all formulations, ranging from −0.95 (A1) to −0.64 (H2), indicating a slight greenish tint. This is typical for rice-based products and may reflect the natural color of the raw materials [24].

The b* values (yellowness) ranged from 5.25 to 6.22, indicating a mild yellow hue. The H-series samples showed slightly higher b* values (5.25–6.22) compared to some S-series samples (5.29–6.12), though differences were relatively small. After cooking, substantial changes in all color parameters were observed compared to raw samples. The L* values of cooked samples decreased significantly across all formulations, ranging from 66.14 to 81.12. This reduction in brightness is attributed to surface gelatinization of starch and potential leaching of soluble components during cooking [20].

The observed color differences among samples can be correlated with their compositional characteristics. The S-series samples, which generally exhibited higher whiteness in both raw and cooked states, correspond to samples with higher protein content (6.47–6.96%) and moderate lipid levels. In contrast, the H-series samples showed lower whiteness, particularly after cooking, which may be related to their lower protein content (6.08–6.34%) and different starch characteristics. H4 showed the most pronounced color changes after cooking, with the greatest reduction in L* value and whiteness, suggesting higher susceptibility to surface gelatinization or component leaching. The color analysis revealed that processing method could significantly affect the appearance characteristics of Tangyuan, with the S-series samples demonstrating superior color stability compared to the H-series. These differences are likely to influence consumer perception and acceptability, as whiteness and brightness are important quality attributes for rice-based products.

### 3.4. Microstructure of Raw and Cooked Tangyuan

**Figure 2:**
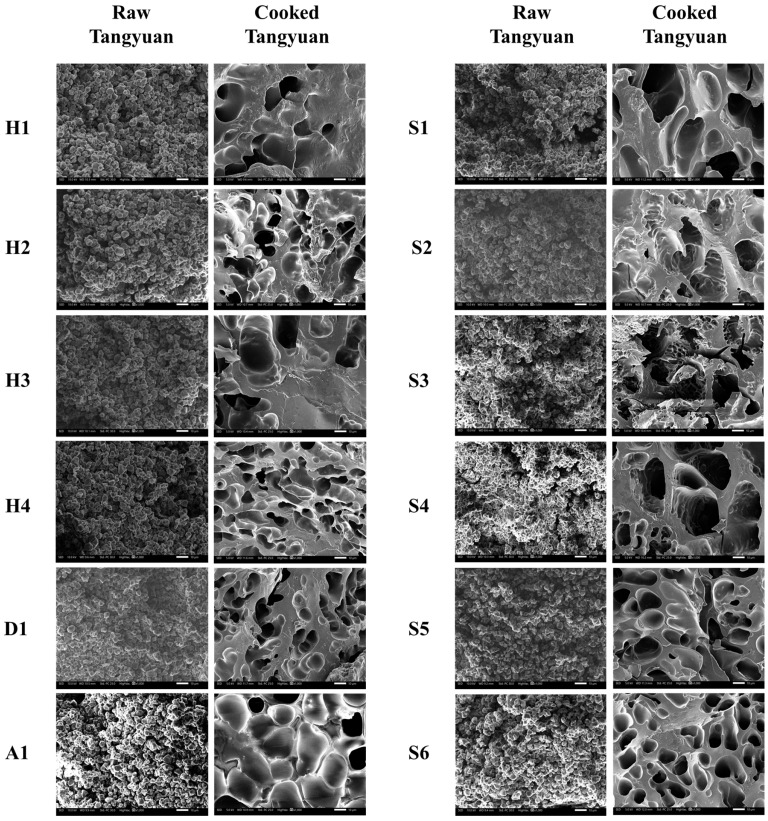
SEM images of raw and cooked Tangyuan prepared by different GRFs (The images were all taken at a magnification of 1000×, with a 10 μm scale bar).

Scanning electron microscopy (SEM) was employed to visualize the microstructural morphology of raw and boiled Tangyuan (Figure 2). In the raw state, all samples exhibited a highly conserved, densely packed granular morphology. The intact, polyhedral glutinous rice starch granules were clearly identifiable, physically embedded within a preliminarily hydrated starch–protein continuous phase. Upon cooking, the microstructure of all samples underwent a dramatic thermodynamic phase transition. The discrete starch granules completely lost their physical identity, melting into a continuous, porous, three-dimensional gel network. This sponge-like honeycomb structure is the microscopic manifestation of starch gelatinization, driven by extensive granular swelling, the loss of semi-crystalline order, and the leaching of amylose chains into the inter-granular space under hydrothermal conditions [25]. However, the architecture of these cooked gel networks varied significantly among the samples, directly correlating with their distinct rheological profiles and macroscopic cooking qualities.

H1 and H3 formed a highly cohesive and dense gel matrix, characterized by thick, continuous pore walls and relatively large, smooth cavities. This robust microstructural integrity indicated a tightly interconnected physical network. Such a dense structural topology aligns well with the high storage modulus observed, endowing the cooked Tangyuan with excellent shape retention, enhanced resistance to shear-induced disintegration, and a desirable chewy texture. S1 and S4 displayed a highly porous, well-interconnected, sponge-like network with notably thinner walls, suggesting a higher water-holding capacity and a more extensive granular expansion during cooking, which typically translates to a softer, waxier sensory bite with lower mechanical hardness [3]. In contrast, S2 and S3 exhibited a severely fragmented, disorganized, and irregular gel matrix. The pore walls in S3 appeared jagged and collapsed, lacking continuous structural support, which implied excessive starch granule breakdown and severe leaching of internal components during the boiling process [26]. Such a compromised, weak network at the microscopic level is the direct mechanistic cause of macroscopic quality defects, specifically high cooking loss (resulting in a turbid soup), structural disintegration, and a mushy texture. These observations confirmed that the inherent physicochemical traits of the selected GRFs dictate not only the rheology of the raw dough but also the ultimate structural stability of cooked Tangyuan. A well-developed, uniform porous network without excessive fragmentation is the microscopic prerequisite for manufacturing high-quality Tangyuan that can withstand the thermodynamic stresses of industrial freezing and consumer boiling.

### 3.5. FTIR and Protein Structure

**Figure 3:**
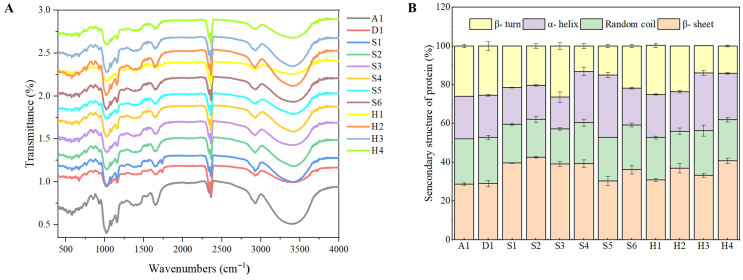
FTIR characterization (**A**) and secondary structure (**B**) analysis of protein.

The Fourier transform infrared spectra of the Tangyuan samples were recorded to investigate the molecular structure and functional group characteristics as shown in Figure 3. The FTIR spectra exhibited characteristic absorption bands corresponding to the major components of the Tangyuan samples, including starch, protein, and lipids. The broad absorption band in the region of approximately 3000–3600 cm^−1^ is attributed to O–H stretching vibrations of hydroxyl groups, primarily from starch and water molecules [27]. The bands around 2925 cm^−1^ are assigned to C–H stretching vibrations of methylene groups, indicating the presence of carbohydrate and lipid components. The absorption region between 1600 and 1700 cm^−1^ corresponds to amide I bands (C=O stretching) and amide II bands (N–H bending), which are associated with protein molecular structure [28]. A qualitative comparison of the relative band intensities among samples showed noticeable variations.

The bands in the region of 1000–1200 cm^−1^ are characteristic of C–O–C stretching vibrations in glycosidic linkages of starch, providing information about starch molecular structure [29]. Differences in the intensity and shape of these bands among samples may reflect variations in amylose/amylopectin ratio and starch crystallinity. For instance, S6, which exhibited the highest amylose content (7.97%), showed distinct spectral features in this region compared to waxy samples (D1, S4) that contained no detectable amylose. The FTIR spectra provided molecular-level insights into the structural characteristics of the Tangyuan samples. These structural features are expected to correlate with the functional properties, including cooking behavior and texture analysis, as discussed.

### 3.6. Textural Properties

**Figure 4:**
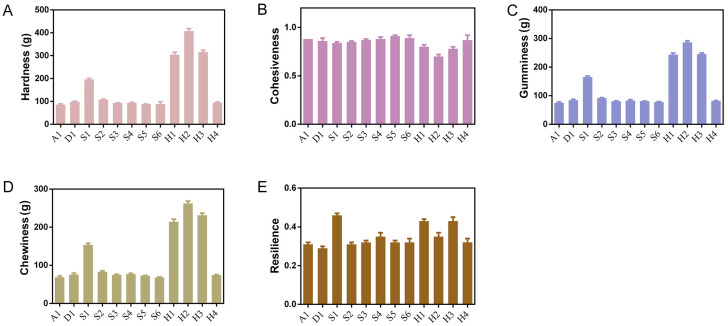
Texture analysis of Tangyuan: (**A**) hardness; (**B**) cohesiveness; (**C**) gumminess; (**D**) chewiness; (**E**) resilience.

The textural properties of Tangyuan samples, including hardness, gumminess, chewiness, and resilience, were evaluated and are summarized in Figure 4. Significant variations in these parameters were observed among different formulations, reflecting differences in their structural integrity. In particular, the samples exhibited distinct hardness values, which are closely related to the cooking quality of glutinous rice dumplings. Higher hardness values suggest a firmer texture, likely attributed to a more compact starch–protein network formed during processing. Such firmness is often associated with reduced water absorption and limited swelling of starch granules during cooking, potentially leading to a denser mouthfeel and longer rehydration time [30]. Hardness is widely regarded as a key quality indicator, primarily influenced by the retrogradation of starch gel, and is affected by the rearrangement of amylose and amylopectin [31].

In contrast, Tangyuan samples with lower hardness, such as A1 and D1, exhibited softer textures, indicating a more fragile structure that may be prone to disintegration during thermal processing. These samples also showed reduced gumminess and chewiness, reflecting lower cohesiveness and structural stability—both critical for maintaining the integrity of glutinous rice dumplings during boiling. Conversely, samples with moderate textural properties, such as A1 and D1, demonstrated a favorable balance between softness and structural stability, which is desirable for achieving a palatable yet stable cooked product. Chewiness, defined as the energy required to chew solid foods [32], as well as hardness, springiness, and adhesiveness serve as key indices for evaluating the palatability of cooked Tangyuan.

Resilience, as a measure of ability to recover after deformation, was also found to vary considerably. A1, D1 and S1 showed higher resilience values, implying better elastic recovery, which is beneficial for preventing collapse during cooking and maintaining a smooth, intact surface after steaming or boiling. Overall, the textural properties presented in Figure 4 provided valuable insights into the relationship between formulation and cooking performance. The results suggest that intermediate levels of hardness, gumminess, and resilience are desirable for achieving optimal cooking characteristics, such as minimal surface cracking, uniform gelatinization, and acceptable sensory quality. Furthermore, well-documented evidence indicated that amylose and amylopectin molecules leach into the surrounding water above the gelatinization temperature, which are likely to contribute to the stickiness of cooked dumplings, thereby directly influencing the cohesiveness values [33,34]. These findings highlight the importance of texture analysis in guiding the development of glutinous rice-based products with improved cooking stability and consumer acceptability.

### 3.7. Size Distribution

**Figure 5:**
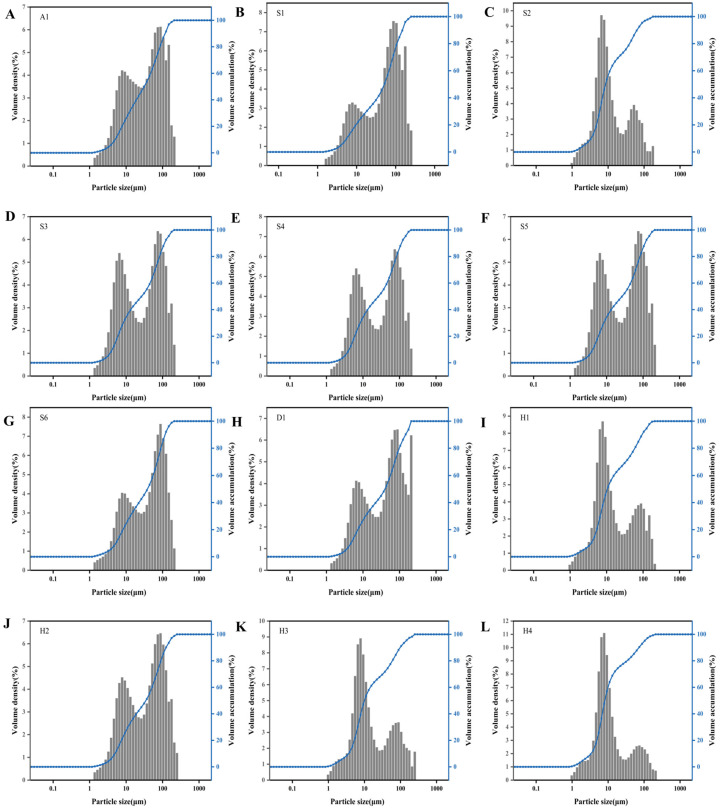
Size distribution of different glutinous rice flours: (**A**) A1; (**B**) S1; (**C**) S2; (**D**) S3; (**E**) S4; (**F**) S5; (**G**) S6; (**H**) D1; (**I**) H1; (**J**) H2; (**K**) H3; (**L**) H4.

The particle size distribution of GRF is a critical parameter influencing hydration kinetics, pasting behavior, and ultimately the textural quality of Tangyuan. All samples showed a unimodal distribution pattern with an average size between 1.0 and 200 μm (Figure 5). S3, S4 and S5 displayed two relatively balanced peaks. Compared to them, S2 and H4 exhibited relatively more uniform size distribution with smaller particle size. The narrow particle size distribution likely resulted from uniform milling conditions, such as controlled wet-milling or jet-milling processes. Coarser flours often exhibit slower hydration rates and lower swelling power due to a lower degree of damaged starch, which might lead to incomplete gelatinization during cooking. Conversely, reduced particle size significantly increases the specific surface area, which strongly correlates with diminished gelatinization temperatures and enhanced hydration properties. The increased water absorption capacity contributes to smoother noodle surfaces and modified textural properties in rice cakes [35,36]. Therefore, flours exhibiting narrow distribution and presence of fines are more recommended for industrial Tangyuan production. If broader distributions of flour must be used, additional classification steps or blending with finer flours may be required to standardize particle size and ensure uniform product quality.

### 3.8. Pasting Properties

**Figure 6:**
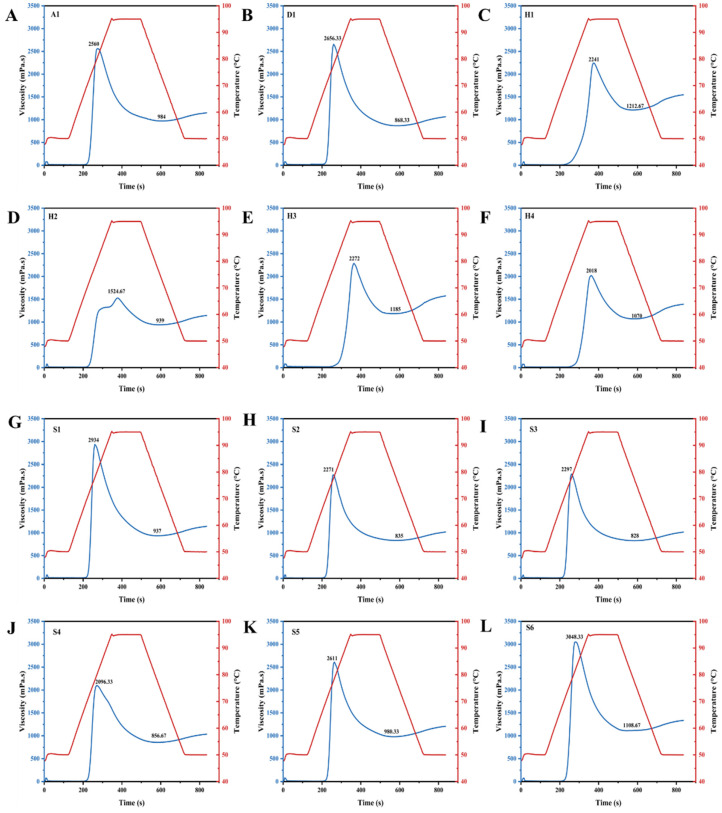
Pasting property analysis of different glutinous rice flours: (**A**) A1; (**B**) D1; (**C**) H1; (**D**) H2; (**E**) H3; (**F**) H4; (**G**) S1; (**H**) S2; (**I**) S3; (**J**) S4; (**K**) S5; (**L**) S6.

The pasting properties of GRF, determined by RVA, are critical indicators of starch granule swelling, thermal–mechanical stability, and retrogradation tendency. As illustrated in Figure 6, the RVA profiles of the 12 samples exhibited pronounced disparities, profoundly impacting their hydration kinetics and suitability for Tangyuan processing. Significant differences were observed in peak viscosity (PV), which reflects the maximum water-binding capacity and swelling extent of starch granules prior to physical breakdown. All samples exhibited typical pasting behavior characterized by an initial increase in viscosity upon heating, followed by a plateau or decline during the holding phase. S6 demonstrated the highest PV (3048.33 mPa·s), closely followed by S1 (2934 mPa·s), indicating highly intact starch granules with robust swelling capabilities. In contrast, H2 exhibited an anomalously low PV (1524.67 mPa·s) accompanied by an atypical, broadened pasting curve with a noticeable shoulder. This restricted swelling in H2 suggested a highly rigid granular structure, which may be attributed to a higher degree of starch damage incurred during milling, or enhanced endogenous lipid–amylose/protein–starch complexations that inhibit water penetration during gelatinization [37].

During the standardized industrial processing of Tangyuan, the dough matrix is subjected to intense mechanical kneading and thermal stress. Therefore, the breakdown viscosity (BD, defined as PV minus trough viscosity) is a crucial parameter reflecting the shear resistance of the swollen granules. Samples with high PVs, such as S6 and S1, correspondingly exhibited substantial BD values (1939.66 mPa·s and 1997 mPa·s, respectively). While high swelling capacity contributes to the desirable “soft and waxy” texture of cooked Tangyuan, an excessively high BD implies that the starch granules are highly fragile and susceptible to disintegration under shear forces. In continuous industrial production, flours with such high breakdown tendencies may result in a hyper-viscous, overly sticky dough matrix. This excessive stickiness severely compromises mechanical machinability, leading to difficulties in filling encapsulation and demolding, ultimately reducing the production yield [22]. Conversely, samples like S4 with PV of 2096.33 mPa·s demonstrated a more balanced rheological profile, offering adequate hydration while maintaining sufficient structural integrity against mechanical shear.

Furthermore, the final viscosity and setback value are primary indicators of short-term starch retrogradation, which is driven by the reassociation of leached amylose and long-chain amylopectin upon cooling. For frozen dough products like Tangyuan, mitigating retrogradation is paramount to preventing quality deterioration during cold-chain storage. As observed in the cooling phase of the RVA profiles, flours that exhibited a steep increase in final viscosity possessed rapid retrogradation kinetics. High setback values directly correlate with the hardening of the Tangyuan dough during freeze–thaw cycles. This matrix rigidification critically diminishes the freeze–thaw stability of the product, resulting in structural collapse, surface frost cracking, and increased cooking loss during terminal boiling [38,39]. Therefore, GRF samples demonstrating moderate peak viscosities alongside low setback values represent theoretically optimal candidates for formulating freeze–thaw-stable, mechanically robust Tangyuan products. The variation among samples highlighted the influence of rice cultivar and milling technique. The high-viscosity group is consistent with waxy rice varieties rich in amylopectin, which tend to form cohesive gels suitable for soft and elastic textures [40].

### 3.9. Rheological Properties

**Figure 7:**
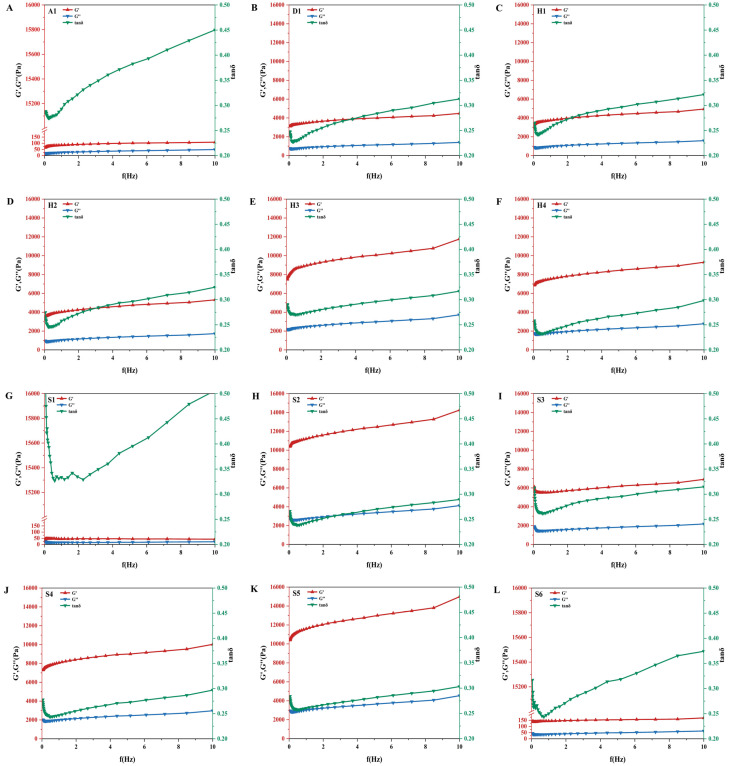
Rheological property analysis of different glutinous rice flours: (**A**) A1; (**B**) D1; (**C**) H1; (**D**) H2; (**E**) H3; (**F**) H4; (**G**) S1; (**H**) S2; (**I**) S3; (**J**) S4; (**K**) S5; (**L**) S6.

Dynamic oscillatory frequency sweep tests were conducted to evaluate the viscoelastic network structures of GRFs. As illustrated in Figure 7, across the entire frequency range (0.1–10 Hz), the storage modulus (G′) was consistently higher than the loss modulus (G″) for all samples. The loss tangent (tan δ = G″/G′) remained below 1.0. These rheological characteristics confirmed that all GRF samples formed a predominantly elastic, solid-like gel network, which is the fundamental structural prerequisite for dough formation and subsequent encapsulation processes [3]. Both G′ and G″ exhibited a slight frequency dependence, indicating that the non-covalent physical cross-links within the starch–protein matrix are transient and susceptible to disruption under higher shear rates.

Although the samples exhibited similar frequency-dependent profiles, the absolute magnitudes of their dynamic moduli varied drastically, revealing profound disparities in their internal network strengths. Based on their G′ values, the samples can be distinctly classified into three rheological categories. Firstly, S2, S5, and H3 formed hyper-rigid networks, displaying exceptionally high dynamic moduli with maximum G′ values exceeding 10^4^ Pa, indicating a highly dense and rigid viscoelastic matrix, likely driven by extensive interactions between starch chains and a robust protein network [41]. Secondly, S3, D1, H1, H2, and H4 formed moderate networks, characterized by intermediate G′ values ranging from 4000 to 9000 Pa, representing a balanced viscoelastic structure. Conversely, A1, S1, and S6 constituted weak and paste-like networks, exhibiting anomalously low moduli. S1, in particular, showed a maximum G′ of barely 50 Pa, coupled with a higher tan δ value (fluctuating around 0.5 at higher frequencies), suggesting a severely weakened, almost paste-like structure with excessive viscous behavior and insufficient elastic recovery.

The rheological properties directly dictated its processing adaptability during industrial Tangyuan manufacturing, specifically affecting kneading, filling encapsulation, and shape retention. Doughs with an excessively low G′ and high tan δ (such as S1, A1, and S6) lack the necessary mechanical strength to support themselves. In an industrial setting, these doughs would be overly sticky, difficult to detach from molds, and prone to structural collapse or tearing during the mechanical injection of fillings (poor machinability) [42]. Notably, A1 exhibited a sharp increase in tan δ at high frequencies within 6.0–10.0 Hz, indicating a transition from gel-like to more liquid-like behavior under high deformation rates.

On the other hand, doughs with extremely high G′ values (such as S2 and S5) possess excellent shape retention but may be overly stiff. While this provides mechanical support, excessive rigidity implies lower extensibility. During the ultra-low-temperature freezing phase, such rigid matrices struggle to accommodate the volume expansion of ice crystals, making the final frozen Tangyuan highly susceptible to surface cracking (frost cleft) [43]. Ultimately, GRF with moderate G′ and G″ values alongside stable tan δ profiles (such as D1 or H1) presents the optimal rheological balance. This well-proportioned viscoelasticity provided sufficient elasticity to encapsulate the filling without collapsing, while retaining adequate viscous flow to ensure smooth mechanical extrusion and buffer the thermodynamic stresses during cold-chain storage. Therefore, specific rheological parameters—particularly G′ at 10–50 Hz and tan δ values below 0.35—can serve as reliable indicators for screening GRF formulations suitable for industrial Tangyuan production.

### 3.10. Digestive Property

The in vitro starch digestibility and subsequent glycemic potential of Tangyuan prepared from different GRF samples exhibited significant differences (*p* < 0.05) as summarized in Table 3. The nutritional quality of starchy foods is fundamentally characterized by their constituent starch fractions: rapidly digestible starch (RDS), slowly digestible starch (SDS), and resistant starch (RS). The starch fraction profiles directly governed the digestion kinetics and the estimated glycemic index (eGI) of the final Tangyuan products.

In particular, sample H1 exhibited the highest RDS value (75.84 ± 0.71%), followed closely by other samples in the H series. Higher RDS levels suggested a more open starch structure or a higher degree of starch damage, leading to rapid enzymatic hydrolysis. In Tangyuan processing, high RDS often correlates with faster cooking times but may result in a more “mushy” mouthfeel. Conversely, A1 and D1 showed significantly lower RDS and higher SDS and RS levels compared to the H series. SDS is digested slowly in the small intestine, providing sustained energy release, while RS escapes digestion entirely. The higher RS in A1 and D1 suggested a more compact crystalline structure or higher amylopectin branch density, which could enhance the structural integrity of Tangyuan, preventing it from collapsing during boiling. The higher RS fraction in certain samples is likely attributable to the reassociation of amylopectin branch chains during cooling. The observed disparities in starch fractions across the samples can be ascribed to a combination of intrinsic structural factors—specifically, the fine structure of amylopectin (e.g., branch-chain length distribution)—and the extent of starch damage sustained during the initial milling process, which dictates the accessibility of enzymes to the starch matrix [44].

The hydrolysis index (HI), which reflects the total glucose released over time relative to a reference food, was most efficiently attenuated in A1, followed closely by S6 and D1. This aligns with the first-order kinetic constant, where variations in the rate of enzymatic hydrolysis dictate the equilibrium concentration (C_∞_). A1 exhibited the lowest C_∞_, highlighting its robust structural resistance to enzymatic breakdown during the simulated digestion phase. The *k* values ranged from 0.048 to 0.070 min^−1^. Samples H1–H4 consistently showed higher *k* values, indicating that their starch is hydrolyzed more rapidly. This rapid digestion is reflected in their higher eGI values, with H1 reaching 89.54. The H series represented high-GI flours (eGI > 85), while the S series (S1–S6) and A1/D1 represent relatively lower GI profiles. For instance, A1 had the lowest eGI (81.01).

## 4. Conclusions

As a quintessential traditional Chinese rice-based food, Tangyuan is widely consumed and holds significant cultural and commercial value. However, similar to other commercially produced frozen convenience foods, industrial Tangyuan is highly susceptible to quality deterioration during prolonged frozen storage. This degradation is primarily driven by physical and physicochemical changes—specifically starch retrogradation and ice recrystallization—which critically compromise its textural integrity, sensory attributes, and overall shelf life. This study comprehensively elucidated the intrinsic structure–function relationships between the physicochemical attributes of multi-cultivar GRF and its processing suitability for industrial Tangyuan. The findings confirmed that the terminal quality and machinability of Tangyuan are not determined by a single parameter, but rather by the synergistic effects of the flour’s microstructure, texture, pasting profile, and dynamic rheological behavior.

Specifically, A1 and D1 demonstrated a favorable balance between softness and structural stability, which is desirable for achieving a palatable yet stable cooked product. Excessive structural damage to starch granules or intrinsically weak physical cross-linking might result in hyper-viscous doughs with poor encapsulation properties. On the other hand, hyper-rigid gel networks combined with high setback viscosities fail to buffer thermodynamic stresses, thereby drastically increasing the risk of structural collapse and frost cracking during rapid freezing and cold-chain storage of Tangyuan products. An optimal processing adaptability necessitates a delicate rheological balance—achieved by GRF exhibiting moderate G′ values and stable tan δ profiles (such as S3, D1, H1, H2, and H4)—which ensured both sufficient elastic support for shape retention and adequate viscous flow for mechanical extrusion.

Beyond processing performance, this study highlighted the immense potential of cultivar-specific screening for nutritional design. Ultimately, constructing a multidimensional suitability evaluation based on these physicochemical traits provides robust theoretical guidance for precision raw material screening, targeted blending, and standardized production, thereby driving the high-quality and health-oriented advancement of the traditional rice-based food industry.

## Figures and Tables

**Table 1:** Approximate composition of different types of GRF.

Samples	Total Starch (%)	Amylose Content (%)	Amylopectin Content (%)	Lipid (%)	Protein (%)	Ash (%)
H1	81.37 ± 0.69 ^f^	1.84 ± 0.04 ^b^	80.09 ± 0.27 ^f^	1.43 ± 0.01 ^d^	6.17 ± 0.13 ^f^	0.27 ± 0.01 ^b^
H2	84.79 ± 0.74 ^a^	2.88 ± 0.05 ^a^	83.42 ± 0.27 ^d^	1.45 ± 0.04 ^d^	6.08 ± 0.10 ^f^	0.26 ± 0.01 ^b^
H3	83.31 ± 0.54 ^d^	1.28 ± 0.04 ^c^	82.68 ± 0.27 ^e^	1.30 ± 0.01 ^e^	6.34 ± 0.13 ^e^	0.30 ± 0.01 ^a^
H4	82.49 ± 0.39 ^d^	2.04 ± 0.02 ^b^	81.81 ± 0.27 ^f^	1.35 ± 0.01 ^e^	6.31 ± 0.12 ^e^	0.30 ± 0.01 ^a^
S1	83.04 ± 0.69 ^c^	0.29 ± 0.05 ^g^	84.31 ± 0.24 ^c^	1.49 ± 0.02 ^d^	6.47 ± 0.17 ^d^	0.22 ± 0.02 ^c^
S2	82.46 ± 0.58 ^d^	0.71 ± 0.03 ^e^	81.78 ± 0.11 ^f^	1.73 ± 0.02 ^b^	6.66 ± 0.13 ^b^	0.22 ± 0.01 ^c^
S3	83.97 ± 0.57 ^b^	1.33 ± 0.02 ^c^	84.69 ± 0.42 ^c^	1.67 ± 0.01 ^c^	6.52 ± 0.16 ^c^	0.17 ± 0.01 ^d^
S4	83.74 ± 0.84 ^b^	0.76 ± 0.04 ^e^	85.91 ± 0.27 ^b^	1.71 ± 0.01 ^b^	6.96 ± 0.29 ^a^	0.14 ± 0.02 ^e^
S5	82.05 ± 0.57 ^e^	0.64 ± 0.03 ^f^	80.34 ± 0.19 ^f^	1.55 ± 0.02 ^d^	6.92 ± 0.13 ^a^	0.16 ± 0.01 ^d^
S6	82.94 ± 0.35 ^c^	0.35 ± 0.02 ^a^	76.85 ± 0.27 ^g^	1.65 ± 0.02 ^c^	6.51 ± 0.12 ^c^	0.16 ± 0.01 ^d^
D1	84.38 ± 0.66 ^a^	1.02 ± 0.02 ^d^	86.38 ± 0.15 ^a^	1.18 ± 0.01 ^a^	6.38 ± 0.23 ^e^	0.29 ± 0.01 ^a^
A1	84.85 ± 0.37 ^a^	0.60 ± 0.03 ^f^	85.50 ± 0.14 ^b^	0.94 ± 0.02 ^a^	6.92 ± 0.17 ^a^	0.25 ± 0.02 ^b^

Note: Means values ± standard deviation, *n* = 3. Different superscript letters in the same row indicate significant differences (*p* < 0.05).

**Table 2:** Color analysis of raw and cooked Tangyuan.

Sample	Raw Tangyuan				Cooked Tangyuan			
	L*	a*	b*	Whiteness	L*	a*	b*	Whiteness
H1	93.14 ± 0.24 ^d^	−0.75 ± 0.06 ^c^	5.25 ± 0.08 ^d^	93.28 ± 0.17 ^d^	69.41 ± 0.12 ^d^	−2.52 ± 0.02 ^d^	3.42 ± 0.03 ^c^	69.54 ± 0.13 ^d^
H2	91.34 ± 0.26 ^f^	−0.64 ± 0.04 ^a^	6.22 ± 0.06 ^a^	91.58 ± 0.14 ^e^	68.62 ± 0.23 ^d^	−2.32 ± 0.04 ^c^	4.79 ± 0.05 ^c^	68.82 ± 0.12 ^d^
H3	93.09 ± 0.14 ^d^	−0.75 ± 0.07 ^c^	5.99 ± 0.04 ^b^	93.30 ± 0.12 ^d^	69.87 ± 0.18 ^d^	−2.15 ± 0.05 ^c^	3.73 ± 0.07 ^c^	70.03 ± 0.12 ^c^
H4	92.92 ± 0.12 ^e^	−0.76 ± 0.02 ^c^	6.09 ± 0.02 ^b^	93.13 ± 0.15 ^d^	66.14 ± 0.15 ^e^	−3.21 ± 0.01 ^e^	2.45 ± 0.01 ^e^	66.24 ± 0.16 ^e^
S1	94.79 ± 0.16 ^c^	−0.83 ± 0.03 ^d^	6.12 ± 0.05 ^a^	95.00 ± 0.18 ^b^	74.48 ± 0.09 ^b^	−3.54 ± 0.05 ^f^	2.86 ± 0.06 ^e^	74.63 ± 0.21 ^b^
S2	94.85 ± 0.08 ^c^	−0.92 ± 0.07 ^e^	5.48 ± 0.08 ^d^	95.02 ± 0.13 ^b^	74.64 ± 0.17 ^b^	−3.63 ± 0.08 ^f^	2.44 ± 0.04 ^e^	74.78 ± 0.18 ^b^
S3	95.13 ± 0.12 ^b^	−0.81 ± 0.01 ^d^	6.12 ± 0.07 ^a^	95.33 ± 0.16 ^d^	75.59 ± 0.15 ^b^	−1.70 ± 0.09 ^a^	8.31 ± 0.01 ^a^	76.06 ± 0.15 ^b^
S4	95.33 ± 0.24 ^b^	−0.67 ± 0.02 ^a^	5.72 ± 0.02 ^c^	95.50 ± 0.21 ^b^	75.62 ± 0.12 ^b^	−1.80 ± 0.02 ^b^	7.18 ± 0.03 ^b^	75.98 ± 0.25 ^b^
S5	96.96 ± 0.16 ^b^	−0.79 ± 0.04 ^d^	5.87 ± 0.07 ^b^	97.14 ± 0.14 ^a^	81.12 ± 0.26 ^a^	−1.73 ± 0.04 ^a^	8.11 ± 0.05 ^a^	81.54 ± 0.14 ^a^
S6	95.75 ± 0.18 ^b^	−0.71 ± 0.09 ^b^	5.29 ± 0.06 ^e^	95.90 ± 0.18 ^b^	73.86 ± 0.18 ^c^	−1.79 ± 0.05 ^b^	7.26 ± 0.08 ^b^	74.24 ± 0.11 ^b^
D1	96.83 ± 0.15 ^a^	−0.72 ± 0.06 ^b^	5.57 ± 0.02 ^c^	97.00 ± 0.23 ^a^	71.43 ± 0.14 ^c^	−1.97 ± 0.03 ^b^	5.07 ± 0.06 ^c^	71.63 ± 0.16 ^c^
A1	94.57 ± 0.18 ^c^	−0.95 ± 0.04 ^e^	5.36 ± 0.09 ^d^	94.74 ± 0.15 ^c^	71.53 ± 0.26 ^c^	−2.57 ± 0.01 ^d^	4.70 ± 0.07 ^c^	71.79 ± 0.13 ^c^

Note: Means values ± standard deviation, *n* = 3. Different superscript letters in the same row indicate significant differences (*p* < 0.05).

**Table 3:** *In vitro* digestibility evaluation of GRFs.

Sample	RDS (%)	SDS (%)	RS (%)	HI (%)	eGI	C_∞_	*k*
**A1**	70.01 ± 0.50 ^f^	26.62 ± 0.39 ^a^	3.37 ± 0.07 ^a^	54.09 ± 0.63 ^g^	81.01 ± 0.24 ^g^	95.28 ± 0.28 ^g^	0.048 ± 0.001 ^g^
**D1**	70.54 ± 0.52 ^f^	26.26 ± 0.20 ^b^	3.20 ± 0.04 ^b^	55.38 ± 0.27 ^f^	81.15 ± 0.16 ^f^	95.41 ± 0.20 ^f^	0.050 ± 0.002 ^f^
**S1**	73.72 ± 0.63 ^e^	24.10 ± 0.39 ^e^	2.18 ± 0.05 ^e^	63.12 ± 0.37 ^c^	84.22 ± 0.25 ^c^	97.06 ± 0.19 ^c^	0.062 ± 0.002 ^c^
**S2**	73.19 ± 0.21 ^f^	24.46 ± 0.18 ^e^	2.35 ± 0.05 ^e^	61.83 ± 0.23 ^d^	82.95 ± 0.27 ^d^	96.79 ± 0.37 ^d^	0.060 ± 0.003 ^d^
**S3**	72.66 ± 0.59 ^g^	24.82 ± 0.26 ^e^	2.52 ± 0.01 ^e^	60.54 ± 0.40 ^d^	82.41 ± 0.19 ^d^	96.51 ± 0.26 ^d^	0.058 ± 0.002 ^d^
**S4**	72.13 ± 0.38 ^h^	25.18 ± 0.15 ^d^	2.69 ± 0.04 ^d^	59.25 ± 0.47 ^e^	81.87 ± 0.43 ^e^	96.24 ± 0.34 ^e^	0.056 ± 0.001 ^e^
**S5**	71.60 ± 0.16 ^i^	25.54 ± 0.43 ^d^	2.86 ± 0.02 ^d^	57.96 ± 0.53 ^e^	81.53 ± 0.25 ^e^	95.96 ± 0.43 ^e^	0.054 ± 0.004 ^e^
**S6**	71.07 ± 0.20 ^bc^	25.90 ± 0.32 ^c^	3.03 ± 0.07 ^c^	56.67 ± 0.70 ^f^	81.29 ± 0.35 ^f^	95.69 ± 0.31 ^f^	0.052 ± 0.003 ^f^
**H1**	75.84 ± 0.71 ^a^	22.64 ± 0.15 ^g^	1.52 ± 0.04 ^g^	68.27 ± 0.31 ^a^	89.54 ± 0.34 ^a^	98.16 ± 0.15 ^a^	0.070 ± 0.002 ^a^
**H2**	75.31 ± 0.39 ^b^	23.01 ± 0.24 ^g^	1.68 ± 0.03 ^g^	66.98 ± 0.37 ^b^	88.13 ± 0.13 ^b^	97.89 ± 0.43 ^b^	0.068 ± 0.001 ^b^
**H3**	74.78 ± 0.67 ^c^	23.37 ± 0.53 ^f^	1.85 ± 0.06 ^f^	65.70 ± 0.44 ^b^	86.82 ± 0.14 ^c^	97.61 ± 0.22 ^b^	0.066 ± 0.003 ^b^
**H4**	74.25 ± 0.45 ^d^	23.74 ± 0.51 ^f^	2.01 ± 0.02 ^f^	64.41 ± 0.60 ^c^	85.51 ± 0.23 ^c^	97.34 ± 0.40 ^c^	0.064 ± 0.001 ^c^

Note: Means values ± standard deviation, *n* = 3. Different superscript letters in the same row indicated significant differences (*p* < 0.05).

## Data Availability

The original contributions presented in this study are included in the article. Further inquiries can be directed to the corresponding author.
